# Using agricultural metadata: a novel investigation of trends in sowing date in on-farm research trials using the Online Farm Trials database

**DOI:** 10.12688/f1000research.26903.1

**Published:** 2020-11-06

**Authors:** Judi Walters, Kate Light, Nathan Robinson

**Affiliations:** 1 Centre for eResearch and Digital Innovation, Federation University Australia, Mount Helen, Victoria, 3350, Australia

**Keywords:** metadata, FAIR, grains, crop, sowing timing, wheat, barley, canola

## Abstract

**Background:** A growing ability and interest in the collection of data, together with the development and adoption of the FAIR guiding principles, has increased the amount of data available in many disciplines. This has given rise to an urgent need for robust metadata. Within the Australian grains industry, data from over thousands of on-farm research trials (Trial Projects) have been made available via the Online Farm Trials (OFT) website. OFT Trial Project metadata were developed as filters to refine front-end database searches, but could also be used as a dataset to investigate trends in metadata elements. Australian grains crops are being sown earlier, but whether on-farm research trials reflect this change is currently unknown.

**Methods:** We investigated whether OFT Trial Project metadata could be used to detect trends in sowing dates of on-farm crop research trials across Australia, testing the hypothesis that research trials are being sown earlier in line with local farming practices. The investigation included 15 autumn-sown, winter crop species listed in the database, with trial records from 1993 to 2019.

**Results:** Our analyses showed that (i) OFT Trial Project metadata can be used as a dataset to detect trends in sowing date; and (ii) cropping research trials are being sown earlier in Victoria and Western Australia, but no trend exists within the other states.

**Discussion/Conclusion:** Our findings show that OFT Trial Project metadata can be used to detect trends in crop sowing date, suggesting that metadata could also be used to detect trends in other metadata elements such as harvest date. Because OFT is a national database of research trials, further assessment of metadata may uncover important agronomic, cultural or economic trends within or across the Australian cropping regions. New information could then be used to lead practice change and increase productivity within the Australian grains industry.

## Introduction

### Digital data

The amount of digital data being generated around the world every day is truly massive. More data were generated between 2015 and 2017 than in the whole length of human history before that ( Marr, 2015). It is expected that the entire digital universe is expected to reach 44 zettabytes ^1^ by 2020 and by 2025, it’s estimated that 463 exabytes ^2^ of data will be created globally each day ( Desjardins, 2019). The sheer volume of data being produced means that excellent data management is essential ( Harper *et al.*, 2018). However, it has been estimated that between 80 and 93% of data are held on personal computers or in offline repositories ( Babcock, 2015), where they are left in the ‘dark’, and are of limited use ( Sadiq, 2016). There are increasing calls for data to be made more widely available for maximum use, as well as the view that research funded by taxpayers should be more readily accessible ( Stow *et al.*, 2018). Research data is no longer just ‘nice to have’: such data underpin decisions about health, development of public policy, innovation, profitability and environmental sustainability ( Barbour, 2019).

### Metadata

For data to be used they need to be brought out of the ‘dark’ and into the ‘light’. That is, they need to be findable. Making data findable is the first step in the ‘FAIR Guiding Principles’ (i.e. Findable, Accessible, Interoperable and Reusable) for scientific data management and stewardship ( Wilkinson *et al.*, 2016), and for data to begin to be considered ‘findable’ they must first be available in digital formation in an online platform (i.e. on the internet). Once online, data are made more findable by having rich metadata. The term ‘metadata’ generally refers to ‘information about information’, or ‘data about data’ ( Brand *et al.*, 2003), and there are increasing calls for metadata to be treated as equally important as the objects they describe ( de Waard & Kircz, 2003). However, metadata records vary greatly in their richness; that is, how much or little of the data is described and captured in the metadata record, where generally the ‘richer the metadata record, the greater the possibilities’ ( Brand *et al.*, 2003).

The term ‘metadata’ can mean different things within different settings, and there are many different ways that metadata can be classified. The ‘Metadata, Encoding and Transmission Standard’ (METS) divides metadata into three broad categories: ‘descriptive’, ‘structural’ and ‘administrative’ ( Davenhall, 2011). Of these, descriptive metadata elements are the most commonly used in outward-facing online searches. For example, putting ‘keywords’ into a search engine such as Google allows sources of online information to be identified and selected as appropriate. Thus, the richer the metadata applied to a data source, the more findable the data becomes.

The creation and use of meaningful metadata are now recognised as crucial elements in providing value-added services ( Simek *et al.*, 2013). Metadata are increasingly being used to detect trends and obtain insights into social, economic and political interactions ( Oh & Park, 2018; Conte *et al.*, 2012; Lazer *et al.*, 2009). For example, many scientific publications have reported use of Google Trends to identify changes in people’s search behaviour as indicators of changing interest in a topic ( Kampf *et al.*, 2015), measures of public health (e.g. Cook *et al.*, 2011), economics ( Kristoufek, 2015) and environmental events ( Cha & Stow, 2015). Such studies have typically relied upon metadata from internet usage or high-throughput data; however, trend detection can be conducted on other types of metadata. For example, metadata from weather stations have been used to detect changepoints that indicate events such as gauge changes or station relocation ( Li & Lund, 2015).

### Accessibility

To maximise use once a data source has been found, the data also need to be accessible. Making data ‘accessible’ is the second step in the FAIR Guiding Principles, meaning that people seeking to use the data can access them at the defined time and by the defined method ( Luque, 2019). Further, there are increasing calls to make research data and findings ‘open’, meaning that data can be ‘used, reused and redistributed freely by any person, and that are subject, at most, to the requirement of attribution and to be shared in the same manner in which they appear’ ( Dietrich *et al.*, 2015). This is especially the case for projects that are publicly funded ( Chugh & Howah, 2019). Thus, the process of and results from experimental research should be open, transparent, reproducible and testable ( Davenhall, 2011). Science funders, publishers and governmental agencies now often require data management and stewardship plans for data that are generated in publicly funded research projects ( Wilkinson *et al.*, 2016). These typically state that data should be published under an ‘open access’ (OA) model. OA is a set of principles and practices through which research outputs are distributed online, free of cost or other access barriers ( Suber, 2004).

### Data repositories

Many types of data and information lend themselves well to OA. For example, many scholarly publishers now provide authors with the opportunity to make the research manuscripts available through OA publishing models, and application of licenses such as those by Creative Commons promote sharing of research outputs. For some types of experimental and research data – particularly those from laboratory-based or sensor-driven experiments – there are a number of well-curated, deeply integrated, special-purpose open data repositories such as Genbank ( Benson *et al.*, 2013), the Worldwide Protein Data Bank ( Berman *et al.*, 2003), and UniProt ( The Uniprot Consortium, 2019). A number of ‘general-purpose’ repositories such as Figshare and DataHub, have also been developed, but not all research data or data types can be captured by or submitted to these repositories, and searching repositories that hold such disparate data is often problematic. Indeed, many potentially valuable datasets emerging from traditional, low-throughput research trials don’t fit well into these repositories ( Subramaniam, 2004).

### Agricultural data

The use of agricultural trial data has enormous potential to improve cropping and management practices ( Hyman *et al.*, 2017). Serra da Cruz & do Nascimento (2019) identified a number of difficulties in data-driven research projects in agriculture, including a lack of appropriate infrastructure to store and preserve data and difficulty in sharing datasets. Harper *et al.* (2018) asserted that ‘the future of agricultural research depends on data’, and that ‘the sheer volume of agricultural biological data being produced today makes excellent data management essential’. These authors also suggest that the ‘value of data increases exponentially when they are properly stored, described, integrated and shared, so they can be easily utilized in future analyses’.

### Grains trials

Within the grains and cropping sector in Australia, many thousands of field-based and on-farm research trials have been conducted by grower and farming systems groups, government researchers, universities and private industry groups with the aim of improving the profitability and sustainability of Australian grain production ( Wills *et al.*, 2019). However, the results from much of this work is traditionally retained ‘in house’ – on personal computers or institutional and private websites ( Serra da Cruz & do Nascimento, 2019) that can be accessed only via subscription or membership status. The data are thus neither findable nor accessible, so the potential value from the re-use of research findings is not being realised. Further, many research topics are being duplicated in both time and space, resulting in wasted time, effort and funding investment ( Sexton *et al.*, 2019).

Identification of this urgent need for greater dissemination of research trial data and findings within the Australian grains research community led to the development of Online Farm Trials (OFT) – an open online database that provides open access to on-farm or field-based cropping research trial data and information. Hosting past and present research trials undertaken and contributed by a range of contributors throughout Australia, OFT is a source of knowledge and information to support decision making, practice change and improvements in farm profitability and sustainability.

OFT can be considered as a ‘biocurator’ ( Harper *et al.*, 2018), striving to present ‘accessible, accurate and comprehensive representation of biological knowledge’. Biocuration is the process of ‘selecting and integrating biological knowledge, data and metadata within a structured database so that it can be accessible, understandable and reusable by the research community’ ( Harper *et al.*, 2018). Data and metadata are taken from trial reports to form the basis of the Trial Projects, which are integrated with other data, including SILO and Bureau of Meteorology weather data and the Soil and Landscape Grid of Australia to deliver a value-added product to database users. OFT Trial Project metadata can be considered as ‘descriptive’, providing information about the basic parameters of each research trial project within the database. The online fields into which mandatory metadata are entered on the OFT website are *Trial project code*, *Trial project title*, *Growing season year*, *Trial site*, *Crop type*, *Trial type*, *Trial design* and *Treatment type*. These fields have been defined as the minimum information metadata elements required for the creation of a Trial Project in the OFT database. On-farm crop research trials typically follow the basic scientific procedure whereby experiments are conducted under controlled, documented conditions, and the results are used to determine the best inputs to achieve the desired outputs. However, this may not be the case for demonstration trials, and scientific publishing standards have not generally been applied within the on-farm research activities in the past, so legacy trial reports do not always contain all the required information to generate searchable metadata within OFT ( Robinson *et al.*, 2019).

## Sowing timing

Sowing time is critical in determining crop yield, so getting the right sowing timing for a crop is one of the most useful ways of maximising grain yield in dryland agriculture ( Sharma *et al.*, 2008). It is generally acknowledged within the Australian grains industry that crops are being sown earlier than in the past ( GRDC, 2011), and it could be expected that cropping research trials would be designed to follow the same practices as those being employed within the general industry to ensure results data are relevant to what growers are doing in their paddocks. However, Stephens & Lyons (1998) suggested that is not always the case, and, to the best of our knowledge, investigations into their claim have not yet been reported.

In the first study of its kind in the grains industry, we investigated whether OFT Trial Project metadata can be used as a dataset to detect trends; testing the hypothesis that research trial sowing dates reflect district practice in grains cropping management (i.e. sowing date moving earlier in the year within the study period).

## Methods

At the time of analysis (18 December 2019), there were 11,458 Trial Projects (i.e. site × growing year × crop type combinations) in the OFT database. These included both published and unpublished trials. Of these, 3634 (30.72%) contained a sowing date (SD) in the available metadata field. Where multiple dates were available (i.e. ‘time of sowing’ trials), the earliest date was used to provide the broadest range of dates being trialled by researchers and to corresponded with the first date used in trials with only a single sowing date. Trial Projects that met the following criteria were included in the analysis of sowing date:

1. winter crop species; specifically, barley, canola, chickpeas, faba beans, field peas, kaspa peas, lentils, linseed, lucerne, lupins, mustard, oats, triticale, vetch and wheat; and
2. sown in an ‘autumn’ period; specifically, between 1 March and 31 July.

The winter crop species selected were those that (i) were contained in records in the OFT database, and (ii) rely on an autumn rainfall ‘break’ to germinate, so would traditionally be sown within a specific ‘sowing window’ aimed at achieving optimal growth and yield. The period between 1 March and 31 July incorporates the broadest possible sowing window for these crop species.

The remaining 3067 Trial Projects were included in the metadata export. The export was saved as an MS Excel spreadsheet, and contained data for ‘Sow date’, ‘Crop type(s)’, ‘Growing season year(s)’ and ‘Trial site(s)’ (i.e. trial location) from the OFT database. Sowing dates were converted from calendar dates to Julian days, the frequency of dates was assessed to determine whether they were normally distributed. All data were found to display a normal distribution, so no data cleaning was required.

Trial Projects sites were located on a map of Australia to show the spatial distribution of trials. Trials in the export were then classified by state (i.e. New South Wales (NSW), Queensland (Qld), South Australia (SA), Tasmania (Tas.), Victoria (Vic.) and Western Australia (WA)) and by crop type (species). There were a limited number of Trial Projects for crop types other than wheat, barley and canola, so data assessments focussed on the six states × three crop types (wheat, barley, canola) and an ‘all crops’ category including all crop types listed above. A total of 24 combinations were generated for analysis.

To determine the minimum number of Trial Projects needed in each state × crop type combination to provide a margin of error (MOE) required for a 95% confidence interval (CI), we calculated the standard deviation of SD across the years on record, then used the following equation to calculate μ, where μ is the sample size of *n* ≥ (z*σ/MOE) ^2^; z* = 1.96 (value corresponding to CI of 95%) and σ is standard deviation of the population. The standard deviation of the sample was 19.5 days, and we selected a MOE of 7 days. From this, a sample size of >29.8 days was calculated. Thus, in the analyses, we included only state × crop type combinations with >30 Trial Projects. SDs were averaged for each year within the remaining state × crop type combination. Ordinary least squares regression plots of SD versus year were created for each of the state × crop type combinations. Linear regression analysis was conducted in StatPlus:macLE build 7.1.1.0 to investigate the relationship between SD and year. The effect of the resultant coefficient of determination ( *R* ^2^) values were considered following Moore *et al.* (2013):

- *R* ^2^ < 0.3 = none or very weak,
- *R* ^2^ such that 0.3 < r < 0.5 = weak,
- *R* ^2^ such that 0.5 < r < 0.7 = moderate, and
- *R* ^2^ > 0.7 = strong.

Plots of residuals versus fitted values were created for each regression to check validity of the assumption of normality in the data. One-way analysis of variance (ANOVA) was used to test the overall significance of the regressions via Student’s *t*-tests. The significance of results was considered: *P* < 0.001, highly significant; *P* < 0.01, moderately significant; and *P* < 0.05, significant; *P* > 0.05, not significant. Plots of residuals demonstrated that the assumption of normality was validated for all regression plots (data not shown).

The sowing date of trials that formed the National Variety Trials (NVT) between 2010 and 2019 were also investigated. An NVT dataset of 6084 trials meeting the same requirements for SD and crop type as utilised above was investigated following the same protocol as specified for the OFT dataset.

## Results and discussion

Metadata is critical to increase the findability of digital information, but it can also be used to detect trends and thus make predictions. Here we used Trial Project metadata from the OFT database as a stand-alone dataset to investigate possible trends in sowing date (SD) of on-farm research trials from across Australia from 1993 to 2019, which was the year range resulting from analysis of the database. The primary purpose of our analysis was to determine if a dataset such as this could be interrogated to provide insights into agricultural trends. In-depth discussions of any agronomic or other factors that could explain specific trends are beyond the scope of the current study.

**Figure 1.**
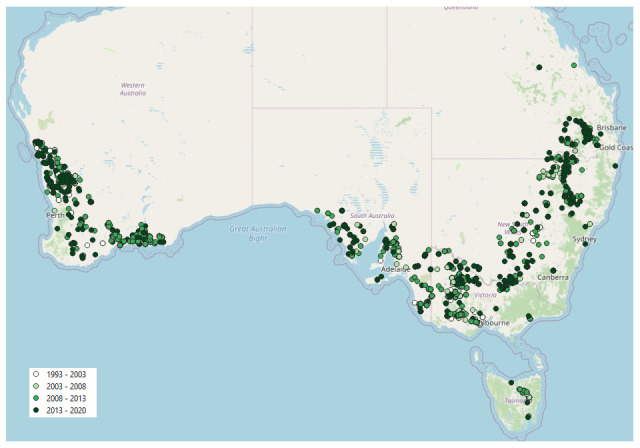
Spatial distribution of Trial Projects from Online Farm Trials used in analysis of sowing date.

The 3067 Trial Projects identified and used in the analysis covered a broad spatial spread of trial sites ( Figure 1) with a similar number of research trials having been conducted in each year across the study period. Our results show that OFT Trial Project metadata can be used to detect trends in SD when sufficient data are accessible. The median SD for all Trial Projects included in the analysis was 140.4 Julian days, which equates to 20 May in a non-leap year (19 May in leap years). The frequency of SDs between designed dates (1 March to 31 July) followed a normal distribution (data not shown), with a standard deviation of 19.5 days. The calculated sample size needed for analysis within each state × crop type combination was 30 Trial Projects, and for the state × crop type combinations that met these criteria, there was only a weak relationship ( *R* ^2^ = 0.25) between the number of SD data points and the *R* ^2^ value of the SD vs year plot, suggesting that 30 data points was sufficient to detect a trend where it existed, and a larger sample size beyond this did not necessarily lead to better trend detection. Similarly, the relationship between the number of years in a state × crop type combination and *R* ^2^ value of the SD vs year plot was also weak ( *R* ^2^ = 0.44), suggesting that a greater span did not always lead to a stronger trend.

Of the 11,458 Trial Projects in the OFT database at the time of analysis, only 3067 contained a record of sowing date (SD) and met the crop-type (i.e. winter crops, see ‘Materials and methods’) and date criteria (i.e. sown between 1 March and 31 July). At the present time, the SD metadata element field in OFT is highly recommended, but is not mandatory because the bulk of Trial Projects in OFT are legacy trials, many of which did not contain a record of SD, or sowing was recorded simply as a period such as ‘late autumn’ or ‘mid-June’, rather than a specific calendar date. These factors limited the number of Trial Projects that could be included in the analyses, and this demonstrates that (i) a record of the SD should be considered mandatory for the reporting of future research trials; (ii) it would be useful for SD to be a mandatory field for current and future Trial Projects from more recent research, and (iii) the format of the date should include a specific date to be references to the Gregorian calendar, which can be converted to a single Julian day if required. Furthermore, we suggest that an international standard should be used to report the date in OFT to increase clarity and interoperability of data. For example, the ISO 8601 standard requires that date and time values are ordered from the largest to smallest unit of time starting with year, month and day, separated by hyphens, e.g. 2020/08/04, meaning the 4th of August 2020.

Analyses of metadata in the OFT database showed that changes in research trial sowing dates over time have differed between the states and crops within the regional cropping areas of Australia. In general, our results suggest that research trial sowing dates in Vic. and WA have been moving earlier each year across the study period ( Table 1), but dates in the other states (SA, NSW and Tas.) do not seem to have changed markedly in the last ~25 years (since ~1993).

**Table 1.** Summary of Trial Projects with sowing date (SD) available in the Online Farm Trials database metadata record with > 30 Trial Projects within a state × crop type combination. (NSW = New South Wales; Qld = Queensland; SA = South Australia; Tas. = Tasmania; Vic. = Victoria; WA = Western Australia).

State × crop type combination ^A^	Trial year range	No. of years in range	No. of trials included	*R* ^2^	*P*-value
NSW ‘all crops’ ^B^	1999–2018	20	399	0.1515	0.099
NSW barley	2001–2017	17	48	0.3771	0.025
NSW canola	1999–2017	19	31	0.0465	0.479
NSW wheat	1999–2018	20	207	0.1917	0.060
Qld ‘all crops’	2006–2015	10	62	0.1587	0.288
Qld wheat	2006–2015	10	32	0.0010	0.946
SA ‘all crops’	2003–2018	16	499	0.3405	0.017
SA barley	2003–2018	16	79	0.3497	0.033
SA canola	2004–2018	15	62	0.2338	0.131
SA wheat	2005–2018	14	223	0.1651	0.189
Tas. ‘all crops’	2000–2014	15	68	0.0301	0.589
Vic. ‘all crops’	1993–2018	26	1053	0.6097	<0.001
Vic. barley	1993–2017	25	226	0.6496	<0.001
Vic. canola	1994–2018	25	145	0.5066	0.001
Vic. wheat	1997–2018	22	441	0.6783	<0.001
WA ‘all crops’	1998–2018	21	978	0.6817	<0.001
WA barley	2001–2018	18	146	0.5997	<0.001
WA canola	1998–2019	22	193	0.8739	<0.001
WA wheat	2000–2018	19	489	0.6431	<0.001

^A^Crops were sown in ‘autumn’, between 1 March and 31 July. ^B^The category of ‘all crops’ included barley, canola, chickpeas, faba beans, field peas, lentils, linseed, lucerne, lupins, mustard, oats, kaspa peas, triticale, vetch and wheat.

Similar trends were undetected in the NVT dataset investigated. No state × crop type combinations had significant changes in SD across years. This is most likely due to the fact that NVT trials are required to be sown during a mandated (specified in trial contracts) sowing window that is deemed appropriate for the crop variety and specific location. SD is therefore predetermined, and is not an independent variable for NVT trials.

One complication in comparing reports of SD lies in the definition of the ‘time of sowing’ (TOS, or ‘sowing date’). Flohr *et al.* (2018) define TOS as ‘the calendar date at which seeds become imbibed and begin the process of germination. For instance, this could be the date on which they are planted into a moist seed bed, or the date on which they receive rainfall/irrigation after being sown into a dry seed bed’. However, we suspect that most reports do not apply this definition, but rather, simply use the date on which the seeds were planted regardless of whether they were dry-sown or how long after the first significant rain (or ‘break’) occurred.

Another complication arises from the observation that choice of sowing date for a crop on a farm is influenced by many factors including climate (especially rainfall events), the size of the cropping enterprise, the equipment and labour available, the tillage method and other management tools to be employed, the crop type and variety to be sown. For research trials, many of these factors are negated, but other limitations may influence the date chosen for sowing. For example, availability of funding, equipment and staff, as well as access to the trial site may play a role in determining the sowing date of a trial. However, these influences are probably usually minor, so likely insufficient to change the desired date significantly. Thus, the SD of a research trial is usually the function of a single establishment date, whereas a sowing schedule on a farm may take anywhere from several days up to a month depending on the size of the area being planted ( Hunt *et al.*, 2019) due to constraints on the availability of machinery and labour ( Fletcher *et al.*, 2016a). In practice, the SD of a research trial can be considered as a distinct entity as the entire trial is usually planted on one day. We suggest it should be compared with the midpoint of farm sowing dates reported elsewhere, which is considered as a good mean measure of whether crops are sown early or late ( Stephens & Lyons, 1998).

### Western Australia

**Figure 2.**
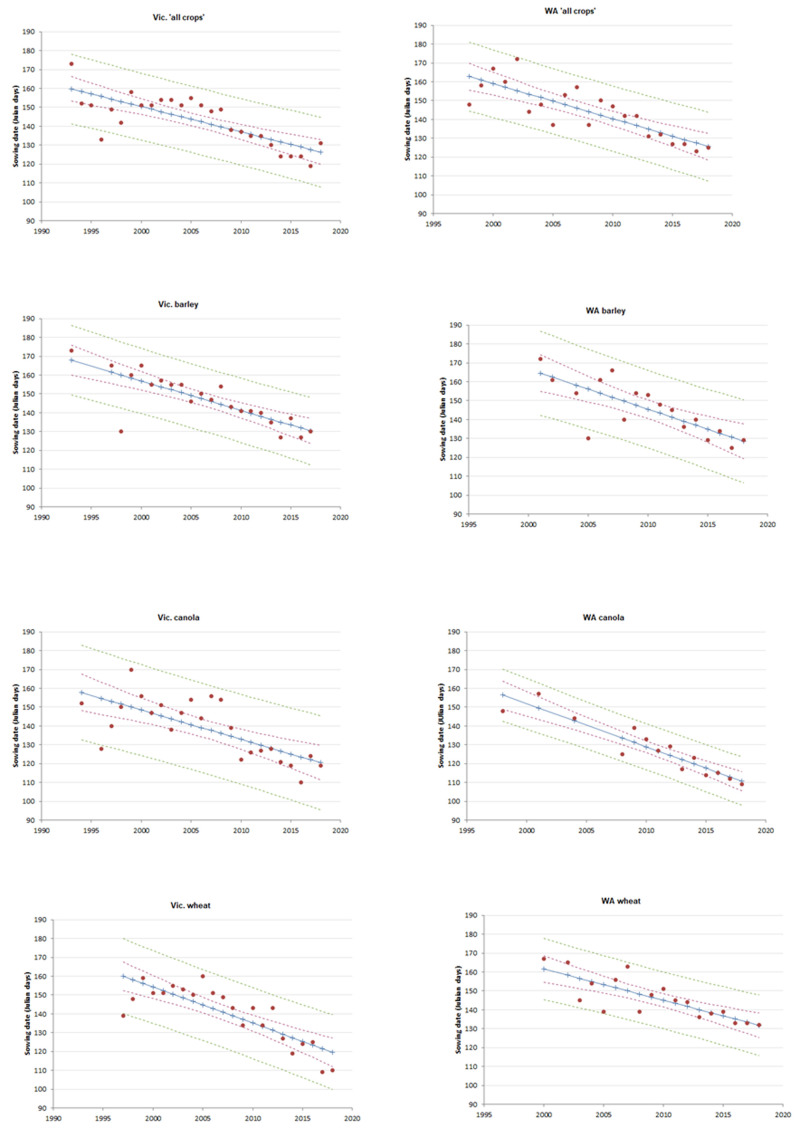
Correlations between mean sowing date and year for state × crop type combinations (>30 trials, *R* ^2^>0.50). The category of ‘all crops’ included barley, canola, chickpeas, faba beans, field peas, lentils, linseed, lucerne, lupins, mustard, oats, kaspa peas, triticale, vetch and wheat. Blue line indicates predicted SD, dashed red lines indicate 95% confidence intervals, and green dashed lines indicate 95% prediction intervals.

The sowing date for cropping research trials for WA SDs, with trials in ‘all crops’ moving earlier by around 1.9 days per year between 1998 and 2018; and wheat, barley and canola trials in WA were sown about 1.7, 2.1 and 2.3 days earlier each year, respectively, for the year analysed in each of these crop species ( Figure 2; Table 2).

**Table 2.** Linear regression analysis of sowing date (SD, Julian days) vs year with records of >30 Trial projects available for that state × crop type combination.

State × crop type combinations	Slope of regression	*P*-value
Vic. ‘all crops’ ^A^	–1.3	<0.001
Vic. wheat	–1.9	<0.001
Vic. barley	–1.6	<0.001
Vic. canola	–1.6	<0.001
WA ‘all crops’	–1.9	<0.001
WA wheat	–1.7	<0.001
WA barley	–2.1	<0.001
WA canola	–2.3	<0.001

^A^‘All crops’ included barley, canola, chickpeas, faba beans, field peas, lentils, linseed, lucerne, lupins, mustard, oats, kaspa peas, triticale, vetch and wheat.

These findings correspond with multiple reports of earlier sowing of crops in general farming practices in WA. Fletcher *et al.* (2016a); Fletcher *et al.* (2016b) reported that field records from seven farms in WA showed sowing of the first cereal crop (wheat or barley) on-farm had advanced markedly in recent years, and was most prominent from 2010 to 2014. The sowing date moved from late May to late April at most sites (although the actual pattern of change was notably different at the seven sites included in the report; see Figure 1 in Fletcher *et al*. (2016b)) and was likely impacted by changes in management and agronomical practices, including adoption of no-till methods and herbicide resistant crop varieties ( Fletcher *et al.*, 2019). This work was based on the report by Stephens & Lyons (1998), who reported that sowing dates of wheat in WA had moved earlier by 1.2 days per year between 1977 and 1990, and confirmed that sowing dates continued to move earlier from around 1995 to 2015. Flohr *et al.* (2018) also confirmed the general shift, reporting that wheat sowing date records from the Yield Prophet database (the online commercialised version of the crop production models APSIM) in WA show a shift of around 1.3 days/year over the 10-year period from 2008 to 2015. Farre *et al.* (2019) asserted that trends in earlier sowing in WA over the last decade also apply to canola crops, and used APSIM-canola simulations to establish the optimum sowing window to maximise grain yield for different locations in WA. A report by DPIRD WA (2019) also noted that ‘in the last decade there has been a trend toward earlier sowing of canola by Western Australian growers’.

Results from the OFT metadata analysis show that sowing of crops in WA research trials reflect the trends seen in general practice in this cropping district, and extends the current knowledge to show that the trend is continuing past 2015, at least as far as 2018, and possibly beyond.

### Victoria

In Vic., the SD for cropping research trials for ‘all crops’ moved earlier by around 1.3 days per year between 1993 and 2018; wheat, barley and canola trials in Vic. were sown approximately 1.9, 1.6 and 1.6 days earlier each year for the years analysed for each of these crop types ( Figure 2; Table 2).. This result is similar to the data from the Yield Prophet database showing a rate of change of 2 days/year between 2008 and 2015 for wheat in Victoria ( Flohr *et al.*, 2018). However, it differs from findings of Stephens & Lyons (1998), whose survey work showed no change in sowing date in the state between 1977 and 1990. These authors note that their data were based on only five survey responses (the least number of any state), so ‘little confidence can be placed on the results’. The OFT metadata suggests that sowing of research trials in Vic. has moved forwards during the study period, in a similar fashion to WA, and thus reflect general practice in cropping across the state.

### Other states

We detected weak trends in SD in three of the state × crop type combinations in these states: NSW barley *R* ^2^ = 0.3771, SA ‘all crops’ *R* ^2^ = 0.3405 and SA barley *R* ^2^ = 0.3497. *R* ^2^ values for all other NSW, Qld, SA and Tas. state × crop type combinations were very weak (< 0.3771), suggesting no clear or consistent relationship between SD and year.

These results differ from those of Stephens & Lyons (1998), who reported that ‘during the 1980s, sowing progressed a day earlier each year at a national scale’. For NSW; however, they note that there were large standard deviations in sowing (wheat) midpoints (see their Figure 4), averaging 21.2 days. Flohr *et al.* (2018) used simulations to show NSW wheat crops were planted 1.1 days/year earlier between 2008 and 2015, but note that southern NSW had the lowest number of fields subscribed to Yield Prophet and that there is a very broad sowing window in this environment. These authors reported sowing of wheat crops in SA has moved 1.3 days/year earlier in the same period. Maitland (2013) reported in the South Australian No Till Farmers Association (SANTFA) newsletter that ‘in recent years, farmers have sown crops earlier in the season’; however, this report contains no data, and thus provides little evidence on which to base further analyses. No published data could be found for Qld or Tas.

### Trend detection

There are several possibilities that could explain why we detected no strong trends in SD in OFT metadata for NSW, Qld, SA and Tas. First, reports of earlier sowing of crops in paddocks may be anecdotal or outdated, and crops were not actually sown earlier in these areas during the period included in our analyses. Stephens & Lyons (1998) surveyed wheat farmers undertaken between 1978 and 1990, which is several years before the earliest record used in our analyses, and almost 20 years before the bulk of the data used here. These authors noted that the national trend towards reduced or minimum tillage techniques coincided with their reported earlier sowing dates, so it is possible that once any farmers who were adopting these different management methods had done so, sowing dates ceased to move any further forwards. The only other reports providing data regarding sowing trends in these states were from the Yield Prophet database, so are for wheat only and derived from simulations rather than measured data. Thus, it is possible that sowing dates for crops in NSW, Qld, SA and Tas. have not changed significantly in the years included in our analyses.

A second possible reason why no notable trends between SD and year were detected for NSW, Qld, SA or Tas., is that research trial SDs in these areas may not reflect general practice in the region, so in fact have not been sown earlier across the study period even if farmers were sowing crops earlier. If the main reason why farmers are sowing crops earlier is increased farm size, then the need for earlier sowing is negated in research trials, meaning they are simply not sown earlier.

Third, it may be that trends in SD exist only within smaller geographic regions within each state, and so have been masked by separate agro-ecological zones. Sowing dates are known to be strongly influenced by geographical regions ( NSW DPI, 2019), driven by variation in a plethora of environmental variables such as rainfall (particularly in autumn; Bloomfield *et al.*, 2018), spring temperatures and frost risk ( Hunt *et al.*, 2019). Large-scale rainfall anomalies have been cited as a driving factor for sowing dates, especially in states with a distinct Mediterranean climate ( Stephens & Lyons, 1998). Frost risk was recently reported to vary considerably across the northern grains region, and manipulation of sowing time was identified as one way to minimise yield losses (in chickpeas) due to frost ( Chauhan & Ryan, 2020). There are likely many reasons that have contributed to this change but investigations into and discussion of the agronomic factors driving earlier sowing are beyond the scope of the current investigation. However, our work demonstrates that OFT provides a useful source of information, and could be used to investigate trends within, for example, different agro-environmental zones or across different rainfall gradients.

### OFT metadata

The OFT database currently provides for the inclusion of exact and accurate geolocation of a trial in the form of latitude and longitude. If entered, this information can be displayed or, for privacy reasons, hidden from the public view at the request of the contributor. Whether hidden or displayed, it can be used to accurately geo-locate a trial site for which climatic variables can be derived for use in analyses. However, few legacy reports contain accurate location information, and even where it may be available, the information is not always entered into the database because it is an optional field. Accurate geo-location (e.g. measurements made via a global positioning system (GPS) could be useful in future analyses, and the location of research trials should be recorded and entered into the database.

The present process of Trial Project creation in OFT is one of manual biocuration, and requires a multidisciplinary effort involving subject area experts, software and technical developers, researchers and project staff. The process of manual biocuration typically involves reading of the trial report and entering data manually into the database. It requires a good understanding of both the research work being entered as well as the functional capacity of the database itself. The original Trial Project entry process for OFT was conducted via a spreadsheet import process, which was managed in-house. Once an upload of projects was completed, the contributor was notified and asked to check that the information had been entered and represented correctly before it was published to the live site (online). However, this process was labour-intensive and slow, and required members of the OFT team to facilitate data entry and publication, so an ‘administration’ centre was developed to allow contributors to enter their data directly to the OFT database without input from the in-house OFT team. This made it easier for contributors to enter data and removed the need for double-handling of trials, however, it simultaneously introduced the problem of quality control. Without the need for Trial Projects to be checked by a member of the OFT team before being published, entry of non-mandatory metadata had not been monitored. Harper *et al.* (2018) note that manual biocuration is perhaps the best way to curate data, but no database has enough resources to curate all data manually. Investments into the Australian grains industry have been recognised as critical drivers for achieving future productivity gains essential for the sustainability and profitability of cropping enterprises ( Walters *et al.*, 2018), so it will be important to evaluate the benefits against the costs of collecting more metadata within the context of ongoing OFT database curation and quality control. There is generally a time investment required to collect metadata, and it is recognised that enriching existing metadata records can be ‘difficult and time consuming’ ( Kemp *et al.*, 2018), so recognition of the trade-off remains an important consideration in the collection of metadata for OFT Trial Projects.

For wheat in particular, the trend of earlier sowing dates may have been facilitated by an increase in use of winter wheat varieties investigated in these areas, as the trend towards earlier sowing is reported to have resulted in the planting of more longer-season varieties and less shorter-season varieties ( Hamblin & Kyneur, 1993; Stephens, 1995). There is currently no metadata field for variety in OFT, thus, the possible role of varietal-driven differences in sowing date trends could not be accounted for in our analyses. Future development of OFT Trial Project metadata to include variety could be highly beneficial in understanding the role of variety in sowing date trends across the different Australian cropping regions.

### Future trends

In our analyses we used simple linear regression, and results suggest that in some areas, research trial crops are continuing to be sown earlier (up to the end of analyses, which was ~2018–19). Simulation studies of wheat in WA suggest that the optimal flowering period (and by extension, sowing date) may move earlier by as much as 29 days under a drier climate ( Chen *et al.*, 2020). This raises the question of how much earlier can crops be planted before the advantage is negated, i.e. how many more years will the current trends persist, and what will be the best way to continue to monitor ongoing shifts in sowing dates in the future to allow for the expected effects of ongoing climate change on crop phenology ( Kukal & Imrak, 2018)? As Trial Projects from current and future research trials are added to the OFT database, further analyses may show further changes in SD trends, and these could be useful in predicting sowing dates to be used during the planning of future research trials. Further, the question of whether earlier sowing in research trials has led to the expected benefits in terms of crop yield has yet to be investigated. At the present time, there is much information in OFT that is not captured in metadata fields, but future development to improve the richness of the metadata would enable these questions, and many others, to be investigated using Trial Project metadata from the OFT database.

### Conclusions

Trial Project metadata from the OFT database is unique in that it can be used in two distinct ways: as filters for online searches of the database; and as a stand-along dataset that can be interrogated to detect trends in recorded fields. Using OFT Trial Project metadata as a dataset we demonstrated that sowing dates of on-farm research trials for ‘all crops’, barley, canola and wheat have moved earlier by 1.3–2.3 days per year from 1993 to 2018 in Vic. and WA. Trends in SD in the other cropping states in Australia were either weak or very weak, suggesting research trials in these areas have not been sown earlier during the study period (1993–2018). To help improve OFT Trial Project metadata for future data discoveries, we recommend that future projects include sowing date as a mandatory field. Numerous other research questions could be investigated using OFT Trial Project metadata, and our work shows that the database provides an effective way for users to access, search, filter and re-use on-farm trials to help improve sustainability and profitability of Australian grains research.

### Data availability

Figshare: Dataset 1: Online Farm Trials Sowing Date Metadata export 18 December 2019 https://doi.org/10.6084/m9.figshare.12895103.v2 ( Walters, 2020a).

This project contains the following underlying data:

- figshare_Dataset 1_Online Farm Trials.xlsx. This dataset was compiled from the Online Farm Trials metadata export on 18 December 2019. It shows the autumn sowing dates (limited to those between 1 March and 31 July) of various crop types across cropping states within Australia between 1993 and 2018. The 'all crops' categories includes barley, canola, chickpeas, faba beans, field peas, lentils, linseed, lucerne, lupins, mustard, oats, kaspa peas, triticale, vetch and wheat.

Figshare: Dataset 2: Online Farm Trials spatial spread of trial sites export 18 December 2019 https://doi.org/10.6084/m9.figshare.12932732.v1 Walters (2020b).

This project contains the following underlying data:

- figshare_Dataset 2_Online Farm Trials.xlsx. This dataset was compiled from the Online Farm Trials export on 18 December 2019. It shows the autumn sowing dates (limited to those between 1 March and 31 July) of various crop types across cropping states within Australia between 1993 and 2018. The data were used to generate a map showing trial site locations within Australia.

Data are available under the terms of the Creative Commons Attribution 4.0 International (CC BY 4.0) Licence.

Readers are also encouraged to visit the Online Farm Trials website where metadata and other information on grain-based trials from across Australia can be accessed ( www.farmtrials.com.au).

## Footnotes

^1^A zettabyte is 1 × 10007 bytes.

^2^ An exabyte is 1 × 10004 bytes.
