# Supplementary material for: Using agricultural metadata: a novel investigation of trends in sowing date in on-farm research trials using the Online Farm Trials database
Source: F1000Res. 2021 May 26;9:1305. Originally published 2020 Nov 6. [Version 2] doi: 10.12688/f1000research.26903.2 (PMC8290206; doi:10.12688/f1000research.26903.2)
Supplement: Peer review history

# Peer review: Reviewer response for version 2

**DOI:** 10.5256/f1000research.56964.r86198

**Published:** 2021-07-19

**Authors:** James Hunt

**Affiliations:** 1 Department of Animal, Plant and Soil Sciences, La Trobe University, Melbourne, Vic, Australia

The authors have attended well to all my suggested changes and I have no further suggestions.

Is the work clearly and accurately presented and does it cite the current literature?

Yes

If applicable, is the statistical analysis and its interpretation appropriate?

I cannot comment. A qualified statistician is required.

Are all the source data underlying the results available to ensure full reproducibility?

Yes

Is the study design appropriate and is the work technically sound?

Yes

Are the conclusions drawn adequately supported by the results?

Partly

Are sufficient details of methods and analysis provided to allow replication by others?

Yes

Reviewer Expertise:

Agronomy, crop physiology, farming systems

I confirm that I have read this submission and believe that I have an appropriate level of expertise to confirm that it is of an acceptable scientific standard.

# Peer review: Reviewer response for version 1

**DOI:** 10.5256/f1000research.29710.r82422

**Published:** 2021-05-04

**Authors:** Medha Devare

**Affiliations:** 1 International Food Policy Research Institute (IFPRI), Montpellier, France

This paper presents the possibility of analyzing metadata to detect trends in agricultural data - using sowing data in on-farm research trials from 1993 to 2019 as a use case to assess if metadata analysis can unearth trends in sowing date. 

 The authors cite FAIR, but in fact, there is nothing in this paper on interoperability and very little on accessibility or reusability - this work is primarily about the value of specifying metadata fields beyond the generic typically requested in standard metadata schemas. However, I wonder how realistic this is - how many fields can be reasonably added to metadata schemas? Aren't NLP and text mining techniques able to get at information to inform this sort of analysis without adding fields to metadata schemas? Data can be richly mined if interoperability using controlled vocabularies or ontologies to describe data variables is implemented by design, surely?

 Nonetheless, this work is interesting and could provide a bit more impetus to improve annotations of research data. It would be good to see more discussion around the possibilities, and how such derivable value could address challenges. I'd focus less on FAIR since this work doesn't really address all aspects of FAIR.

 OFT seems like an interesting resource, but I was unable to export data that was open - and not all of the data in the db is - so that needs to be better clarified in the paper. I wanted to see more on the metadata schema used, the decisions that went into this, how it was received by researchers/data managers, etc. I would suggest a bit of a rewrite to explore these angles, leading into the particular use cases - which are good.

 One nitpicky point: The authors say "More data were generated between 2015 and 2017 than in the whole length of human history before that ( Marr, 2015)" Presumably Marr was predicting more data being generated... in 2015, and not stating that it was so!

 I also don't quite get the 3067 and 3634 numbers in the methods - and how they relate to the 11,458 projects. I also don't understand why the notion of "trial project" is needed - it is confusing. Aren't these just all trials that meet the filters of site, growing year, and crop type?

Is the work clearly and accurately presented and does it cite the current literature?

Yes

If applicable, is the statistical analysis and its interpretation appropriate?

I cannot comment. A qualified statistician is required.

Are all the source data underlying the results available to ensure full reproducibility?

Yes

Is the study design appropriate and is the work technically sound?

Yes

Are the conclusions drawn adequately supported by the results?

Yes

Are sufficient details of methods and analysis provided to allow replication by others?

Partly

Reviewer Expertise:

agronomic research for development, data management, OA, FAIR, responsible data, semantic web

I confirm that I have read this submission and believe that I have an appropriate level of expertise to confirm that it is of an acceptable scientific standard, however I have significant reservations, as outlined above.

# Response

**Published:** 2021-05-18

**Authors:** Judi Walters

**Affiliations:** Federation University Australia, Australia

We note the reviewer’s comment that there is “ nothing in this paper on interoperability and very little on accessibility or reusability [and that] this work is primarily about the value of specifying metadata fields beyond the generic typically requested in standard metadata schemas”. This is a reasonable comment with which we do not disagree, but would add that the paper does not claim to discuss all elements of FAIR. Rather, it mentions FAIR to place the discussions around the benefits of the OFT database in a broader context of the publication and re-use of research data. Then, the paper highlights the value of using the metadata fields used in OFT as a way to search for information, much like a bibliographic database of research papers.

 The reviewer asked “how many fields can be reasonably added to metadata schemas?” and suggest that “NLP and text mining techniques able to get at information to inform this sort of analysis without adding fields to metadata schemas?” We suggest that, although some researchers may be able and willing to go to the lengths required to using text mining techniques to locate information, there are many researchers (particularly in the agricultural sciences), agronomists, and grain growers, for whom this type of searching would not be possible. Hence, the value of the simple, easy, and freely-accessible searches made available via OFT.

 The observation that “[d]ata can be richly mined if interoperability using controlled vocabularies or ontologies to describe data variables is implemented by design, surely?” is true, and work in progress may mean that OFT adopts a controlled vocabulary to increase interoperability in the future.

 We have included some changes in response to other reviewers, so hope this satisfies the suggestion for further discussion around research data annotation/use.

 Without further clarification we are unable to determine the reasons why the reviewer was unable to “open export data”, and suggest that contact be made with the OFT team via email ( oft@farmtrials.com.au) to get support because all exports in the database should be retrievable with sufficient internet capabilities.

It was decided to avoid further discussion of the metadata schema used because further development under the current funding contract is expected to explore this aspect of OFT in the near future, and subsequent publications would be better to address this topic in greater depth.

We thank the reviewer for picking up the extrapolation error when that we said "More data were generated between 2015 and 2017 than in the whole length of human history before that” and given the citation was Marr (2015) presumed that Marr was predicting more data being generated. We checked the reference and note that Marr wrote “The data volumes are exploding, more data has been created in the past two years than in the entire previous history of the human race”, so we have corrected the text to reflect this.

To clarify the numbers “3067 and 3634” in the “Methods”: of the total number of trial projects in the OFT database at the time of investigation (i.e. 11,458), 3634 trial projects had data available for sowing date (SD). Of the 3634 trial projects that had SD available (and could therefore be included in the investigation), 567 trial projects had SDs that were outside the dates considered reasonable for an ‘autumn’ sowing period (i.e. not between 1 March and 31 July), so that left 3067 trial projects available for the investigation.

The concept of a ‘trial project’ is unique to OFT and was so named to define a database entry for a site x growing year x crop type combination. The institution of ‘trial projects’ was necessary so that an individual piece of research work that spanned a multiple of any one of these factors could be entered in the system with adequate metadata so it could be located via a metadata search.

# Peer review: Reviewer response for version 1

**DOI:** 10.5256/f1000research.29710.r82540

**Published:** 2021-05-04

**Authors:** James Hunt

**Affiliations:** 1 Department of Animal, Plant and Soil Sciences, La Trobe University, Melbourne, Vic, Australia

This manuscript describes a study in which metadata from the Online Farm Trials website were used to detect changes over time in trial management, specifically sowing date. The analysed results are compared to published data relating to grower sowing date and the differences at a state level and possible causes are discussed. This exercise is used as an example of how agricultural metadata can be used to detect trends in management practice.

 The manuscript is well written and well prepared and will be of interest to agricultural researchers.

 It should be noted that grower time of sowing data are available in the following peer reviewed references, which are stronger sources of evidence than the GRDC fact sheet that is cited under the heading ‘Sowing timing’.

 Flohr, BM, Hunt, JR, Kirkegaard, JA, Evans, JR, Trevaskis, B, Zwart, A, Swan, A, Fletcher, AL, Rheinheimer, B (2018) Fast winter wheat phenology can stabilise flowering date and maximise grain yield in semi-arid Mediterranean and temperate environments. Field Crops Research 223, 12-25. ^1^

 Anderson, WK, Stephens, D, Siddique, KHM (2016) Dryland Agriculture in Australia: Experiences and Innovations. In 'Innovations in Dryland Agriculture.' (Eds M Farooq, KHM Siddique.) pp. 299-319. (Springer International Publishing: Cham) ^2^

 [I note the Flohr *et al.* (2018) reference is cited later in the manuscript]

 Both references use the same data source (the Yield Prophet® database, see Hochman, Z, van Rees, H, Carberry, PS, Hunt, JR, McCown, RL, Gartmann, A, Holzworth, D, van Rees, S, Dalgliesh, NP, Long, W, Peake, AS, Poulton, PL, McClelland, T (2009) Re-inventing model-based decision support with Australian dryland farmers. 4. Yield Prophet® helps farmers monitor and manage crops in a variable climate. Crop and Pasture Science 60, 1057-1070.). ^3^ The Anderson *et al. *(2016) reference also uses the Stephens & Lyons (1998) data. The Flohr *et al.* (2018) reference breaks the trends down to a state level which would make a useful comparison to this study.

 Crop types – ‘Kaspa’ is a cultivar of field pea, not a distinct species, and experiments with this cultivar can be included in the field pea category.

 Normality of data - do you mean no data transformations were required (rather than data cleaning, which implies removal of data)?

 I don’t think the definition provided by Flohr *et al.* (2018) re imbibed seeds provides a problem for his study because growers are sowing earlier regardless of the timing of rainfall (i.e. ‘dry’ sowing). See;

 Fletcher, AL, Robertson, MJ, Abrecht, DG, Sharma, DL, Holzworth, DP (2015) Dry sowing increases farm level wheat yields but not production risks in a Mediterranean environment. Agricultural Systems 136, 114-124. ^4^

 Fletcher, A, Lawes, R, Weeks, C (2016) Crop area increases drive earlier and dry sowing in Western Australia: implications for farming systems. Crop and Pasture Science 67, 1268-1280.# ^5^

 Fletcher, A, Flohr, BM, Harris, F (2019) Evolution of early sowing systems in southern Australia. In 'Australian Agriculture in 2020: From Conservation to Automation.' (Eds J Pratley, JA Kirkegaard.) pp. 291-305. (Agronomy Australia and Charles Sturt University: Wagga Wagga)

 It would be worth noting that rainfall of sufficient magnitude to germinate seed (the ‘autumn break’) is arriving later and that this could be having an effect on results. See;

 Flohr, BM, Ouzman, J, McBeath, TM, Rebetzke, GJ, Kirkegaard, JA, Llewellyn, RS (2021) Redefining the link between rainfall and crop establishment in dryland cropping systems. Agricultural Systems 190, 103105. ^6^

 Pook, M, Lisson, S, Risbey, J, Ummenhofer, CC, McIntosh, P, Rebbeck, M (2009) The autumn break for cropping in southeast Australia: trends, synoptic influences and impacts on wheat yield. International Journal of Climatology 29, 2012-2026. ^7^

 In the paragraph headed ‘Other states’, the statement that Flohr *et al. *(2018) used simulation to show NSW wheat crops were planted 1.1 days/year earlier between 2008 and 2015 is incorrect. The reported shift in sowing dates are actual dates entered by growers into Yield Prophet® i.e. they are actual grower sowing dates, not simulated.

 Likewise in the paragraph headed ‘Trend detection’ the following statement is incorrect:

 “ *The only other reports providing data regarding sowing trends in these states were from the Yield Prophet database, so are for wheat only and derived from simulations rather than measured data*.”

 These data ARE measured observations, not simulated. This misinterpretation needs to be corrected. Albeit the sample of growers in the Yield Prophet database is biased toward early adopters of technology.

Is the work clearly and accurately presented and does it cite the current literature?

Yes

If applicable, is the statistical analysis and its interpretation appropriate?

I cannot comment. A qualified statistician is required.

Are all the source data underlying the results available to ensure full reproducibility?

Yes

Is the study design appropriate and is the work technically sound?

Yes

Are the conclusions drawn adequately supported by the results?

Partly

Are sufficient details of methods and analysis provided to allow replication by others?

Yes

Reviewer Expertise:

Agronomy, crop physiology, farming systems

I confirm that I have read this submission and believe that I have an appropriate level of expertise to confirm that it is of an acceptable scientific standard, however I have significant reservations, as outlined above.

## References

[1] : Fast winter wheat phenology can stabilise flowering date and maximise grain yield in semi-arid Mediterranean and temperate environments. *Field Crops Research* .2018;223: 10.1016/j.fcr.2018.03.021 12-25 10.1016/j.fcr.2018.03.021

[2] : Dryland Agriculture in Australia: Experiences and Innovations.2016; 10.1007/978-3-319-47928-6_11 299-319 10.1007/978-3-319-47928-6_11

[3] : Re-inventing model-based decision support with Australian dryland farmers. 4. Yield Prophet® helps farmers monitor and manage crops in a variable climate. *Crop and Pasture Science* .2009;60(11) : 10.1071/CP09020 10.1071/CP09020

[4] : Dry sowing increases farm level wheat yields but not production risks in a Mediterranean environment. *Agricultural Systems* .2015;136: 10.1016/j.agsy.2015.03.004 114-124 10.1016/j.agsy.2015.03.004

[5] : Crop area increases drive earlier and dry sowing in Western Australia: implications for farming systems. *Crop and Pasture Science* .2016;67(12) : 10.1071/CP16200 10.1071/CP16200

[6] : Redefining the link between rainfall and crop establishment in dryland cropping systems. *Agricultural Systems* .2021;190: 10.1016/j.agsy.2021.103105 10.1016/j.agsy.2021.103105

[7] : The autumn break for cropping in southeast Australia: trends, synoptic influences and impacts on wheat yield. *International Journal of Climatology* .2009;29(13) : 10.1002/joc.1833 2012-2026 10.1002/joc.1833

# Response

**Published:** 2021-05-18

**Authors:** Judi Walters

**Affiliations:** Federation University Australia, Australia

We agree with the reviewer’s suggestion that reference to grower time of sowing data available in Flohr et al, (2018) and Anderson et al. (2016) provide stronger sources of evidence than the GRDC fact sheet that is cited under the heading ‘Sowing timing’, so have added in-text citations to these references alongside that of the GRDC fact sheet.

 We note that the crop type ‘Kaspa’ is a cultivar of ‘Field pea’, rather than a distinct species, but because the OFT database separates these we have decided to keep them separate in this work.

 The reviewer asked in relation to ‘normality of data’ whether we mean no data transformations were required (rather than data cleaning, which implies removal of data): we confirm no data transformations were performed, neither were any data removed other than as described in the ‘Materials and methods’.

 We note the comment regarding the definition provided by Flohr et al. (2018) re imbibed seeds not providing a problem for his study because growers are sowing earlier regardless of the timing of rainfall (i.e. ‘dry’ sowing), so have retained the text and added a further comment including reference to Fletcher et al. (2015, 2016a, 2019).

 We have also noted that rainfall of sufficient magnitude to germinate seed (the ‘autumn break’) is arriving later and that this could be having an effect on results; including reference to Pook et al. (2009) and Flohr et al. (2021) as suggested.

 We have corrected the error in the paragraph headed ‘Other states’, stating that Flohr et al. (2018) used simulation to show NSW wheat crops were planted 1.1 days/year earlier between 2008 and 2015, now saying that the reported shift in sowing dates are actual dates not simulated data.

 The paragraph headed ‘Trend detection’ has also been corrected to reflect that ‘actual’ rather than ‘simulated’ nature of data from previous reports, and include a note from the reviewer (pers. comm.) that “the sample of growers in the Yield Prophet database is biased toward early adopters of technology”.

# Peer review: Reviewer response for version 1

**DOI:** 10.5256/f1000research.29710.r74582

**Published:** 2020-12-10

**Authors:** Sjaak Wolfert, Daoud Urdu

**Affiliations:** 1 Wageningen University & Research, Wageningen, The Netherlands; 2 Wageningen Economic Research, Wageningen University & Research, Wageningen, The Netherlands

- This study aims to explore the possibility of using and analyzing metadata in order to tackle overarching agricultural challenges like yield prediction. By applying statistical methods for a specific question, the value of metadata is presented. Different literature has been studied and categorized as part of the introduction. However, the interrelation between these data categories and FAIR could be more clear in the introduction part. Also, the objective could be stated more clearly.
- Furthermore, the reviewers think that the statistical part could be reviewed by someone with a more statistical background.
- In the context of FAIR – This paper seems to contribute to the Accessibility part. However, this could be stated more explicitly. The other characters (F, I, and R) in the abbreviation could also get some more attention.
- With regard to standardization – what is the role of data modelling and information modelling? What does the study contribute to the interoperability part of FAIR? Is ISO8601 the only relevant standard?
- Two figures 3067 and 3034 were used. This brought a slight confusion. 

Is the work clearly and accurately presented and does it cite the current literature?

Partly

If applicable, is the statistical analysis and its interpretation appropriate?

I cannot comment. A qualified statistician is required.

Are all the source data underlying the results available to ensure full reproducibility?

Partly

Is the study design appropriate and is the work technically sound?

Yes

Are the conclusions drawn adequately supported by the results?

Yes

Are sufficient details of methods and analysis provided to allow replication by others?

Yes

Reviewer Expertise:

Information Modelling, Systems Analysis, Digital Innovation

We confirm that we have read this submission and believe that we have an appropriate level of expertise to confirm that it is of an acceptable scientific standard, however we have significant reservations, as outlined above.

# Response

**Published:** 2021-05-18

**Authors:** Judi Walters

**Affiliations:** Federation University Australia, Australia

The Introduction of this paper introduces the category of digital data and links the amount of digital data being produced with the need for it to be both ‘findable’ and ‘accessible’ to be of increased use. The role of metadata in making digital data more findable is then discussed. The accessibility of data is then also discussed in the context of open access publishing models. The other components of the FAIR guiding principles (i.e. interoperability and reusability) are not discussed in detail as they were considered outside the scope of the current work: constituting another entire conversation in their own right. For this reason, we have chosen to leave the text as it stands.

 The reviewer requested that the objective be stated more clearly, so we changed the wording in the second paragraph under ‘Sowing timing’ to clarify.

 We note the reviewer’s comment that the statistical part could be reviewed by someone with a more statistical background, and confirm that we did consult a statistician at Federation University during the analysis phase of the research. We were advised that further analysis techniques would not add to the main objective of the work: to demonstrate that metadata can be used to detect trends, rather than to apply highly-sophisticated statistical methods to prove or disprove a specific hypothesis, which, we agree, would necessitate greater statistical rigour if that had been our objective. Because statistical complexity was unnecessary to demonstrate the information we were trying to convey we did not attempt to complicate the data with extra analysis.

 The reviewer is correct that “In the context of FAIR – this paper seems to contribute to the Accessibility part”, so we have highlighted this further by adding a sentence to this effect at the beginning of the Conclusions.

 As stated above, the I and R components are not given greater attention because we consider them to be separate discussions, beyond the scope included here.

 The reviewer asked what is the role of data modelling and information modelling?

 The reviewer asked “What does the study contribute to the interoperability part of FAIR?”. We reiterate that the study was not attempting to focus on Interoperability, and that the comment regarding the ISO 8601 was given as an example of what could be implemented in the future to increase interoperability of OFT Trial Projects. The ISO standard mentioned is not the only relevant standard but is provided as an example only.

 The reviewer’s comment regarding ‘slight confusion’ around the numbers 3067 and 3034 was investigated, and we suspect the reviewer was referring to ‘3634’ rather than ‘3034’. For clarification, 3634 refers to the number of Trial Projects containing sowing date in the metadata field, and this number was reduced to 3067 by the limits set on the sowing window (1 March to 31 July).
